# Growth and development in prefecture-level cities in China

**DOI:** 10.1371/journal.pone.0221017

**Published:** 2019-09-03

**Authors:** Daniel Zünd, Luís M. A. Bettencourt

**Affiliations:** 1 Mansueto Institute for Urban Innovation, The University of Chicago, Chicago, IL, United States of America; 2 Ecology and Evolution, The University of Chicago, Chicago, IL, United States of America; 3 Santa Fe Institute, Santa Fe, NM, United States of America; University of Lausanne, SWITZERLAND

## Abstract

Nowhere has the scale and scope of urbanization been larger than in China over the last few decades. We analyze Chinese city development between the years 1996 and 2014 using data for the urbanized components of prefecture-level cities. We show that, despite much variability and fast economic and demographic change, China is undergoing transformations similar to the historical trajectory of other urban systems. We also show that the distinguishing signs of urban economies—superlinear scaling of agglomeration effects in economic productivity and economies of scale in land use—also characterize Chinese cities. We then analyze the structure of economic change in Chinese cities using a variety of metrics, characterizing employment, firms and households. Population size estimates remain a major challenge for Chinese cities, as official numbers are often reported based on the Hukou registration system. We use the information in the residuals to scaling relations for economic quantities to predict actual resident population and show that these estimates agree well with data for a subset of cities for which counts of total resident population exist. We conclude with a list of issues that must be better understood and measured to make sense of present urban development trajectories in China.

## Introduction

China has transformed itself in just a few decades from a rural, low-income nation to a booming urban system, displaying rates of economic development and social change that are unprecedented in history for a nation of its size. As recently as the mid-20th century, China’s economy was dominated by its primary sector and urbanized areas provided only a minor part to the value creation of the national economy. The vast majority of the population lived in rural areas, accounting for an urbanization rate of only 20% as recently as in 1975 [1]. Within a few decades this picture changed completely: by 2011, more than half of the Chinese population lived in urban areas and the contribution of the primary sector to the national economy in terms of value became almost negligible.

The explosive growth of Chinese cities and the general demographic and economic restructuring of the country via massive urbanization are the principal manifestations of this monumental transformation [2]. Certain quantities, such as GDP, land area, and road length [3, 4], follow scaling laws also in the Chinese urban system. However, relatively little is known quantitatively about the development trajectory of Chinese cities among other quantities on the aggregate. Specifically, whether their growth and development have parallels in past historical examples in other nations and show similar patterns of agglomeration, as urban theory would suggest.

To investigate these issues, we provide here a comprehensive attempt at characterizing Chinese urbanization, by analyzing the patterns of growth and transformation of mainland China’s top nearly three hundred prefecture-level cities. Prefecture-level cities are a main administrative unit in China, one rank below that of provinces (the highest non-national level administrative unit). They consist of several districts and counties, which are classified as rural or urban depending on their economic and demographic character. In the following, we will use the term “prefecture-level city” and “city” to denote the collection of districts and counties in each prefecture-level city that are classified as urban. Although this is not an ideal definition of a functional urban area—such as Metropolitan Areas in the US and Europe [5], which are defined at finer spatial scales based on integrated economic activity and commuter flows—it is a workable, consistent definition that approximates the same unit of analysis given extant Chinese statistics. Note, however, that while this is true for larger cities, the present definitions do not extend to smaller scales [6]. Except for some important special cases, such as the Pearl River delta, these units of analysis are clearly separable from each other spatially and thus may be regarded as clear, geographically contiguous urbanized areas, without any other prefecture-level cities close by.

One of the peculiar challenges of understanding Chinese urbanization has to do with how population counts are organized according to the nation’s official statistics. Among other difficulties [6], one of the main challenges with the count of people is that the Chinese government classifies its citizens into two categories, rural or urban residents by the *Hukou* system. A person’s Hukou status is defined at birth, mainly based on their family history [7]. Citizens with a family history in one of the prefecture-level cities generally have an urban Hukou, which allows them to move freely between all prefecture-level cities and enjoy subsidized amenities and social benefits across the urban system. These include access to education and work, among other socioeconomic services [8]. Citizens with a rural Hukou are not entitled to these same urban benefits but enjoy others, such as the possibility to own land.

At present, only local governments can change their citizens’ Hukou status. Thus, the current resident classification system provides a tool to control the migration of rural citizens into urban areas and the mobility of the population in general. This control mechanism makes it possible for local authorities to suppress, or at least substantially discourage, the emergence of uncontrolled growth of informal settlements for the poorest in China, as seen in many other fast urbanizing countries [9], but also foregoes the possibility of naturally attracting and integrating rural populations in cities and increase the nation’s urbanization rate at a faster pace. The system itself has experienced substantial change over time and is likely to be reformed in the future [10, 11].

For many authors, the ability to control population migration has been seen as one of the main factors for the unprecedented success of China’s economic policies over the last few decades [12]. At the same time, this method of controlled migration, and informal responses to it, rises questions of equity, the kind of urbanization China is experiencing and how it compares to historical examples and present theory [13–16].

In terms of its consequences for understanding urbanization, the Hukou system of resident classification introduces several empirical difficulties when analyzing data for Chinese cities. Official China City Statistical Yearbooks produced by the National Bureau of Statistics of China and prefecture-level cities only typically include population estimates for those with a local Hukou status. Typically, permanent residents with an urban Hukou from other cities are not counted locally but rather in their city of original registration. The same issue affects residents with a rural Hukou, who are not counted in any of the prefecture-level cities, only at their county of registration. Only recently have a few prefecture-level cities started to perform a true census of their full resident population. These estimates make it possible to develop and evaluate a method to estimate the true size of some of the larger and more attractive Chinese cities based on the magnitude of their scaling effects. It allows us to check estimated population sizes in a sample of cities with universal counts and extrapolate our predictions to some of the more interesting cases where no such data are available.

Before getting to this issue at the end of the manuscript, we start with a description of the system of Chinese prefecture-level cities and existing data from official statistics. A number of interesting quantities are available annually at this scale that characterize urban economies on the aggregate, in terms of sectors, financial accounts, conditions of households and firms, and indicators of added value (profits). We also use these data to characterize the longitudinal transformation of the Chinese urban system and demonstrate that its cities share a number of well known general agglomeration effects, i. e. superlinear scaling with city size, the tell tale of urban economies and observed for most systems of cities in the world [17–19]. This section looks at two most prominent quantities, the Gross Domestic Product (GDP, i. e. the size of urban economies) and the built up area for the same cities. We show that, despite a relatively large level of variation from city to city, both quantities scale on average in line with theoretical expectations and with observations from other urban systems. The next section takes this analysis a step further and looks more closely at a number of economic quantities that characterize the Chinese urban economy and their relations to scaling. As we shall see, existing empirical data presents us with a number of difficulties and uncertainties. We identify several aspects of Chinese urban data that seem to us inconsistent given urban theory and the historical trajectory of other urban systems. We conclude with an outlook for the future of Chinese cities and the kind of empirical evidence that would be desirable to create a much more stringent scientific analysis of their progress and the possibility of a rigorous comparative analysis to other urban systems throughout the world.

## Scaling & agglomeration effects in Chinese prefecture-level cities

The tell tale signal of cities and urbanization is a set of general phenomena known as scaling relations in geography and complex systems, and as agglomeration effects in economics [20]. The simplest empirical expression of these phenomena manifests itself as the statistical tendency of larger cities within an urban system to create increasing returns to scale in their economic performance and economies of scale in their land uses and infrastructure.

More specifically, larger cities tend to have larger economies (measured e. g. by their Gross Domestic Product) on a per capita basis than smaller towns. They also tend to be denser spatially, for example, as measured by the amount of land that is built up as infrastructure and places of work and residence. Moreover, the behavior of these expected values for each city given its population size is scale-invariant and does not depend on a particular city size, and, thus, is well described by power-law functions.

To see this more explicitly in the case of Chinese prefecture-level cities, we write any urban extensive quantity, *Y*_*i*_(*t*), such as GDP or land area, for city *i* at time *t* as

$$\begin{array}{c} Y_{i}\left(t\right)=Y_{0}\left(t\right)N_{i}{\left(t\right)}^{\beta }e^{\xi_{i}\left(t\right)}, \end{array}$$ (1)

where *Y*_0_(*t*) is the intercept characterizing the baseline quantity per capita in the smallest cities in the system, *β* is the scaling exponent (or elasticity, in the language of economics), *N*_*i*_(*t*) is the city’s population, and *ξ*_*i*_(*t*) is a residual to the scaling relation.

Eq (1) is exact, and a general parameterization of *Y*_*i*_. The advantages of this form, however, derive from the fact that *β* is a general quantity, which tends to be approximately independent of city size *N*_*i*_ and takes specific values *β* = 1 ± *δ*, (with the theoretical value, *δ*^*T*^ ≃ 1/6) [20]. In such case, Eq (1) becomes very simple, a scale invariant (power-law) function of city size, while the residuals of the logarithmic fit are

$$\begin{array}{c} \xi_{i}\left(t\right)=\text{ln}\;Y_{i}\left(t\right)-\beta \;\text{ln}\;Y_{0}\left(t\right)N_{i}\left(t\right). \end{array}$$ (2)

Note that by definition of residuals they are independent of city size, *N*_*i*_, and are zero on average, in the sense that $\sum_{i=1}^{N_{c}}\xi_{i}\left(t\right)=0$, where *N*_*c*_ is the number of cities in the urban system, so that the magnitude of the *ξ*_*i*_(*t*) expresses how well a city performs relative to others in the urban system. For this reason the *ξ*_*i*_ are sometimes known as Scale-Invariant Metropolitan Indicators, where the word *Metropolitan* refers to the functional definition of cities (metropolitan areas) as mixing socioeconomic networks embedded in space, in the sense of urban theory.

**Fig 1:**
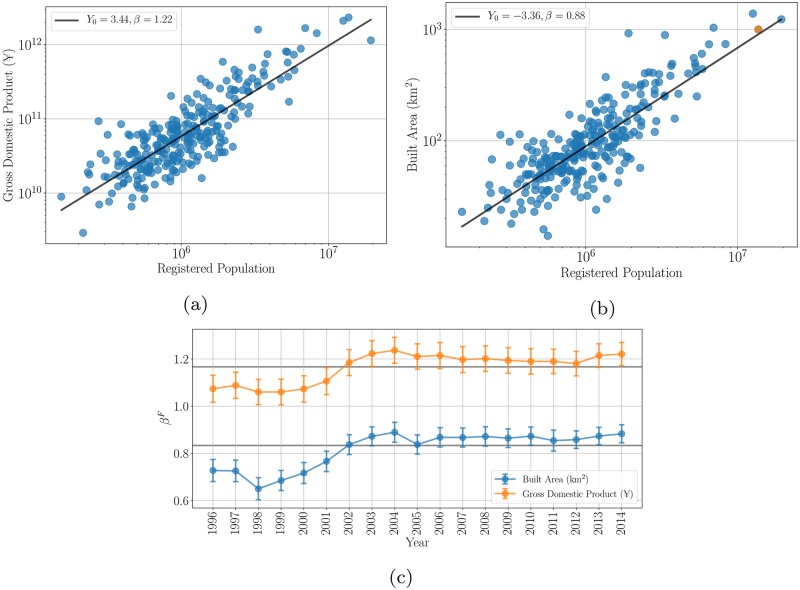
Scaling relations and exponents for Chinese prefecture-level cities. The superlinear scaling law for the Gross Domestic Product vs. size by population in 2014 (a). The exponent *β* is 1.22 (95% CI [1.171 1.269]). The sublinear scaling law for the built up surface vs. size by population in 2014 (b), with an exponent *β* of 0.88 (95% CI [0.842 0.918]). No data for Shanghai is provided for 2014, the orange symbol is the built up surface of Shanghai in 2013. Temporal evolution of the exponents for GDP and built area with the respective 95% CIs (c). The two gray lines depict the simplest expectation for the theoretical exponents at 7/6 and 5/6, respectively. These exponents clearly show increasing returns to scale in economic performance and economies of scale in land use.

Fig 1 illustrates the nature of scaling effects for Chinese prefecture-level cities, using GDP and built up surface. We see that, as broadly expected from theory, the fitted exponent characterizing GDP is larger than one ($\beta_{GDP}^{F}≃1.22>1$) while that for built area is smaller than one ($\beta_{Area}^{F}≃0.83<1$). However, the results are rather noisy and confidence intervals tend to be larger than in other nations, such as the United States. The effect is robust and persists over time, as Fig 1c depicts.

**Fig 2:**
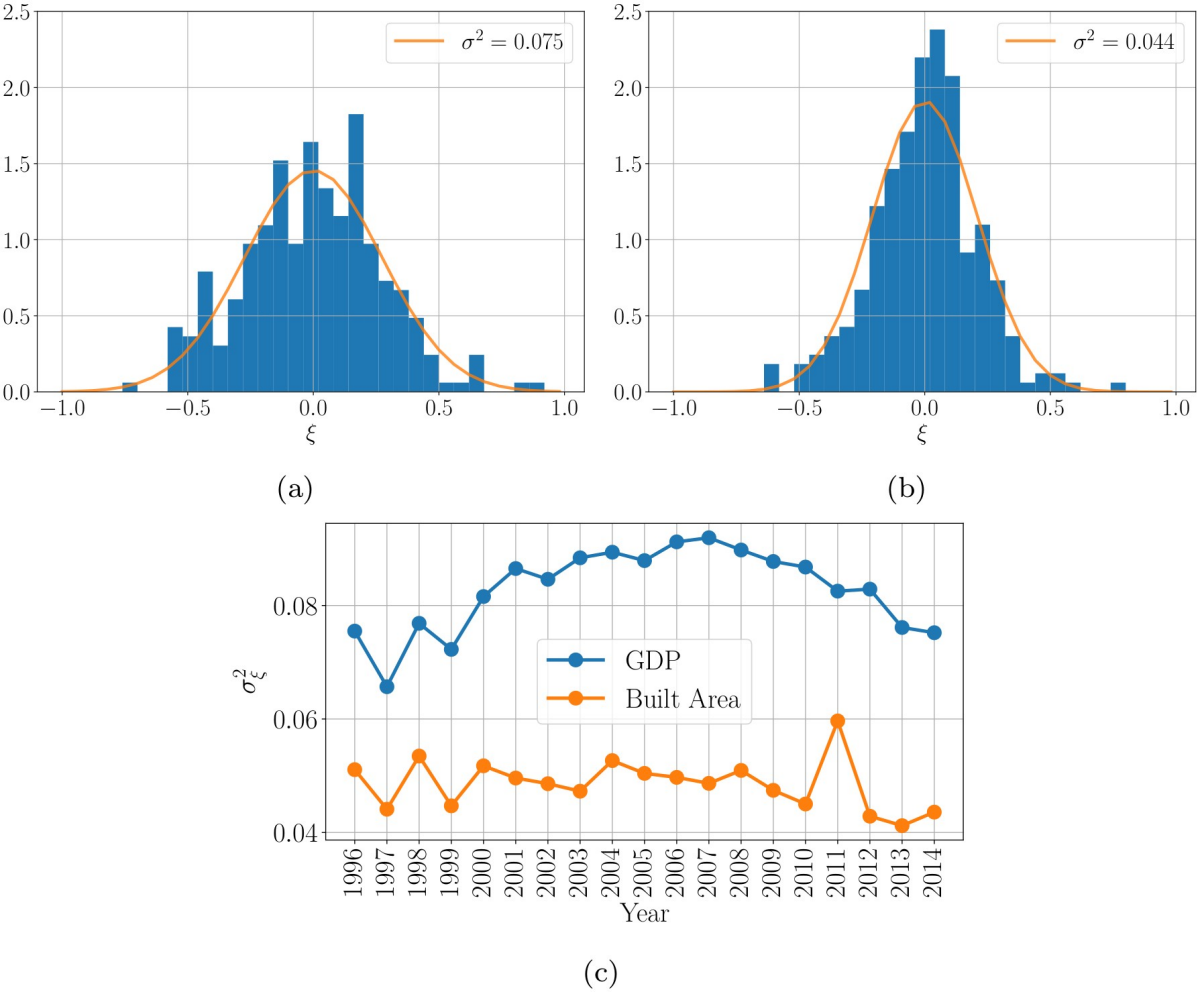
Histograms showing the frequency distribution of the residuals for the Gross Domestic Product of Prefecture-level cities (a) and their built up surface area (b) in the year 2014. The distributions can be roughly described by a Normal distribution (orange line). (c) depicts the temporal evolution of the variance *σ*^2^ from the respective distribution of the residuals. The distribution of residuals has a relatively large variance *σ*^2^ compared to other urban systems.

**Fig 3:**
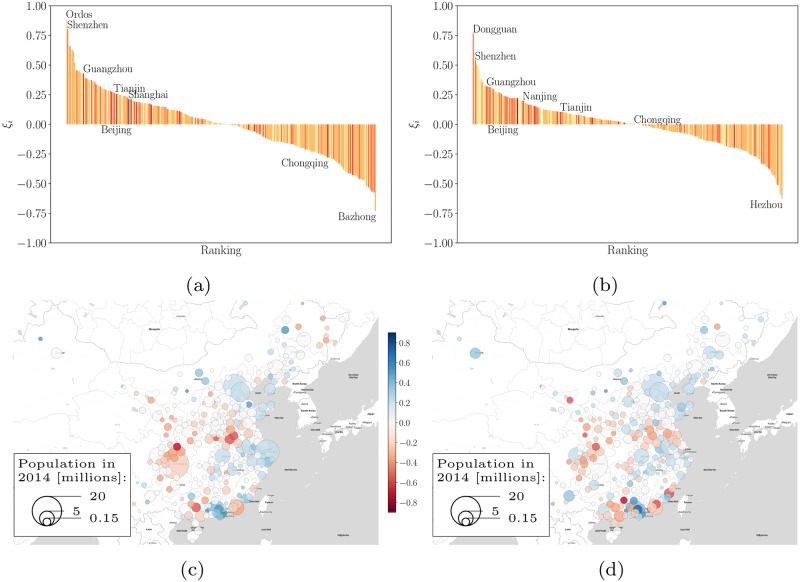
Local measure of urban dynamics and reference scale for ranking Chinese cities, the symbol areas are scaled according to the registered population. Scale independent ranking of cities in terms of Gross Domestic Product (a) and built area (b). Spatial distribution of the scores from the scale independent measure [21]: GDP (c) and built up surface area (d). The ranking shows that large coastal cities tend to overperform for China in the scale independent ranking.

Because of relatively large deviations from the average scaling trend in Chinese prefecture-level cities (see Fig 2), the analysis of the residuals *ξ*_*i*_ is particularly interesting. Fig 3 shows the distribution of residuals, and a map of China showing their magnitude.

In terms of GDP, we see several notable and relatively well known large deviations from scaling. For example, Shenzhen—specializing in the manufacture and export of advanced electronic products and adjacent to Hong Kong—is the most exceptional large city in the nation for its GDP. It is only surpassed by the relatively small city of Ordos, which is rich in natural resources—especially coal—and has been the subject of substantial central government investment, leading to the highest GDP per capita in China.

Generally speaking, many large cities in China deviate from the scaling noticeably, mostly by over performing in terms of GDP. Among the largest cities, only Chongqing, nominally the largest prefecture-level city in China, appears too poor for its size, as well as too large spatially. This points to the fact that its spatial definition—an enormous region covering more than 82 thousand km^2^—is probably a bad approximation to the actual size and extent of its functional city.

In terms of land area, we see a similar pattern as for the GDP, but with smaller deviations from scaling (see Fig 2c). Most of the rich cities in terms of GDP per capita—e. g. Shenzhen, Beijing—also exhibit a strong deviation from scaling in terms of built up area.

In conclusion, while there is a large amount of variability among Chinese prefecture-level cities, average scaling shares similar properties to other urban systems. It shares a similar magnitude for observed scaling effects and is broadly in line with urban scaling theory [17, 20, 22, 23].

In this respect, we note that not only is $\beta_{GDP}^{F}$ somewhat larger than the expected theoretical value ($\beta_{GDP}^{F}>\beta_{GDP}^{T}≃7/6$), but also that many large cities display large positive Scale-Invariant Metropolitan Indicators for GDP, which are known to be attractive to internal migrants outside the Hukou system. Both empirical effects are potentially partly explained by the fact that the population numbers used in this section for measuring scaling are sometimes underestimates of the real resident population. A larger putative population for these cities would indeed move these points to the right, towards the scaling line and would potentially decrease its slope. We explore this possibility as a means of predicting actual resident population in Chinese cities in a later section. Before we do so, we will show that the economic performance of Chinese prefecture-level cities, as measured by a number of distinct indicators characterizing households, firms and local governments, largely mirrors the scaling behavior of overall GDP.

## Economic facets of Chinese prefecture-level cities

In the previous section, we showed that Chinese prefecture-level cities exhibit increasing returns to their population scale in terms of their reported GDP; despite a substantial city to city variability and some issues of potential bias concerning population estimates. Nevertheless, the scaling exponent is roughly in line with theoretical expectations and observations of many other urban systems worldwide [17, 20, 22, 23]. In this section, we explore this issue further, by taking a closer look at a number of quantities characterizing the economy of Chinese cities from the perspective of households, firms and the finances of local governments.

**Fig 4:**
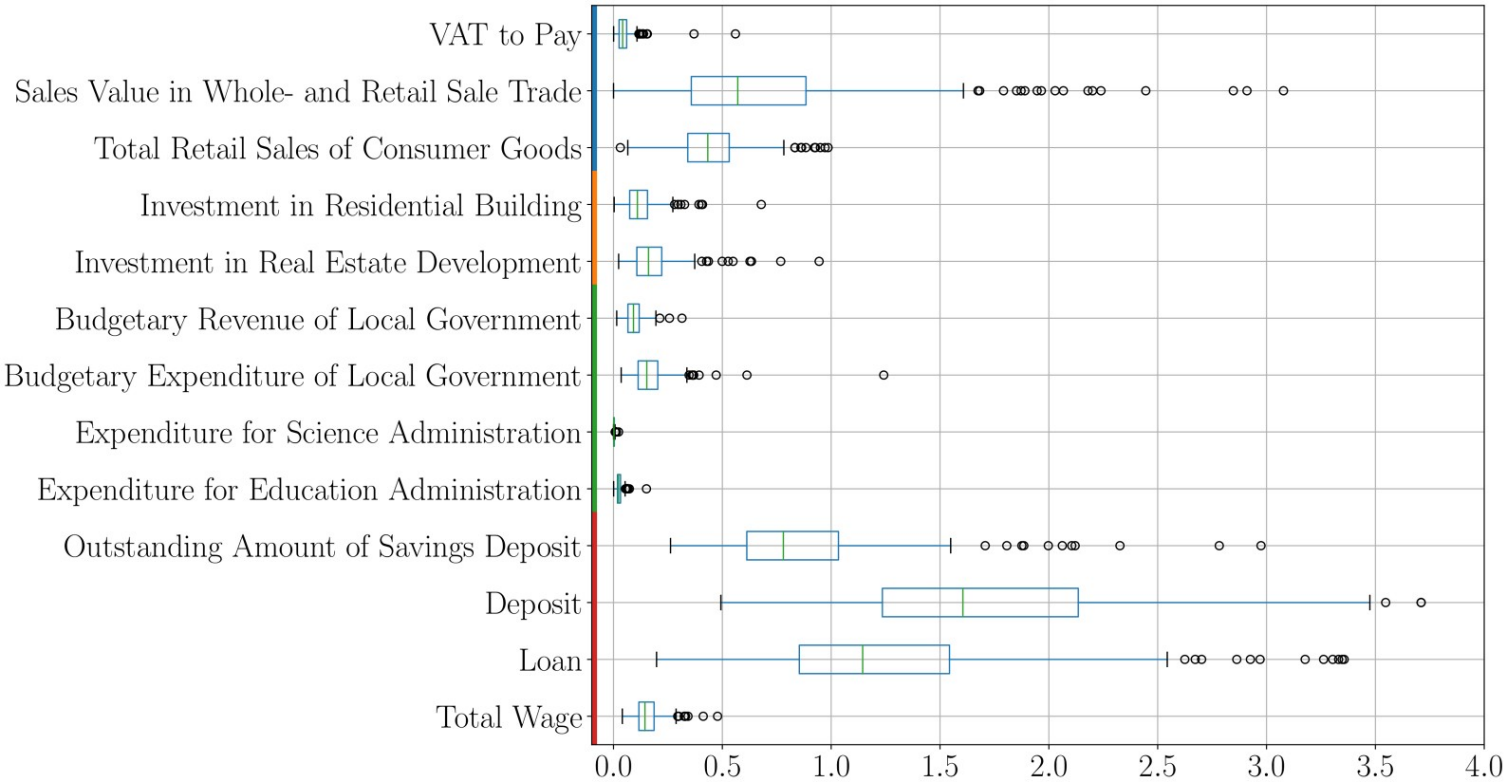
Boxplots of the ratios of different economic quantities characterizing prefecture-level cities in China in relation to their urban Gross Domestic Product. These economic quantities can be divided into four categories: general economy (blue), investments in real estates (orange), budgets of prefecture-level governments (green), and financial behavior of households (red). These quantities show higher variabilities than corresponding GDP, which is a more aggregated measure.

Fig 4 summarizes a number of distinct economic quantities and their relationship to local GDP. Note that some of these quantities, such as bank deposits and wages in state-owned companies are local flows, i. e. money per unit time, and should be expected to be a fraction of local GDP. Others, such as wholesales, may in part represent export flows between cities, while still others, like government revenues, may be the result of local governance and taxes. Clearly these quantities are very different not only in terms of their meaning, but also their magnitudes.

**Fig 5:**
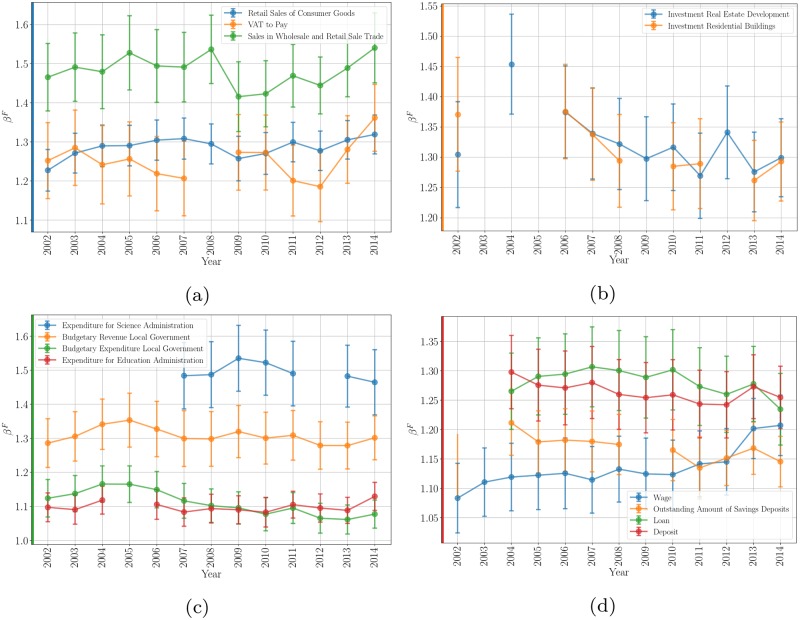
Temporal evolution of the scaling exponent for different economic quantities provided by the statistical offices of Chinese prefecture-level cities: General economy (a), investments in real estates (b), budgets of prefecture-level governments (c), financial behavior of households (d). The high variability of the quantities is manifested not only in between cities, but also over time.

Fig 5 shows the scaling exponents for a number of interesting quantities reported by Chinese Statistics for its prefecture-level Cities. These quantities fall into four classes, characterizing the general economy, investments in real estate, the budgets of prefecture-level governments and the financial behavior of households.

All these quantities show even larger variability, depicted by the large error bars, than more aggregated quantities such as GDP. This effect is not only present between cities, but also from year to year. In many cases, clear from the several panels in Fig 5, data are only available for a subset of years, creating a rather fragmentary picture. Nevertheless, we see that all exponents are superlinear (*β*^*F*^ > 1), and that the corresponding 95% confidence intervals remain above unity in all cases. Thus, a large variety of economic quantities representing business activity, real estate investment, local government finances and household behavior, *all* are characterized by strongly superlinear effects with population size within the Chinese urban system.

A few more particular issues are reveled by a closer analysis of these results. Regarding the general economy, we see that value added tax (VAT), a measure of the profits of companies, is roughly in line with the agglomeration effects observed for GDP. Retail sales, however, show a somewhat stronger exponent, suggesting that larger cities may provide tradable sales exported to smaller places and abroad. This effect—as measured by the corresponding scaling exponent—is even stronger for wholesales. Investments in real estate, as shown in Fig 5b, also show somewhat stronger agglomeration effects relative to GDP, a phenomenon that is common to other urban systems as a result of concomitant average increase in land use density with city size [20]. These measurements are subject to large uncertainties though, as shown in Fig 5b.

The budget revenue of local governments appears as a slightly growing fraction of GDP with city size—indicated by a large scaling exponent similar to real estate investments– which is also consistent with VATs collected, at least over the last few years. Some science expenditures (described as Science Administration) show the strongest concentration in larger cities, with largest scaling exponent in Fig 5c, a phenomenon possibly due to central government investment and transfers, which also echoes strong superlinear effects of patents in the USA [19].

Finally, the behavior of households suggests that the economy of larger cities is characterized by both greater value of deposits and access to credit per capita, roughly by the same degree. Interestingly, the exponent on savings is superlinear too, but weaker, indicating that wealth accumulation may lag behind rates of economic activity. In recent years, these numbers would suggest that a decreasing fraction of GDP with city size is being saved by Chinese households, perhaps a signal of increasing cost of living in larger cities.

Wages, in Fig 5d, reflect only those of state-owned enterprises. It is interesting that the exponent characterizing these seems to be increasing over time, signifying that the labor productivity of state-owned enterprises increases substantially in larger cities. In the last few years, the value of these exponents suggests that wages of state-owned enterprises are approximately a constant fraction of local GDP, regardless of city size. This may be in line with the most recent economic reform when most of the SOEs, except the most profitable, were privatized (see S1 File), and the changes in ownership structures following the establishment of the China State-owned Assets Supervision and Adminstration Comission in 2003, which might have introduced rent of monopoly for SOEs.

In summary, all quantities characterizing many different aspects of the economies of Chinese cities show strongly superlinear effects, clearly indicating that the wealth and productivity of China as a nation is strongly predicated on its urbanization and in particular on the economic agglomeration effects of its (largest) urban areas.

## Estimating true resident population

We have seen, in the previous sections, that the way of counting population in Chinese cities raises issues of biases and accuracy. The Hukou system creates situations where highly desirable cities will have their populations typically underestimated while at source, in other cities and rural areas, population must be correspondingly overestimated.

Only recently have some of the prefecture-level cities started to perform a true census of their full population. In 2014, several cities generated this kind of data and published them in the China City Statistical Yearbook, specifically the directly-controlled municipalities Beijing, Shanghai, Tianjin, and the provinces Guangxi, Guangdong, and Hainan. All of those provinces are located in the coastal areas of China, which have experienced the largest rates of growth, thus, the data might be biased to a certain degree.

In the following, we will show how the scale-invariance of the mean GDP for a city given its population size can be used to estimate the true population of Chinese cities in a simple way.

It is well known, that the population of Beijing, for example, is largely underestimated by almost 40%, when only residents with a local Hukou are counted [24]. This difference can be described by a factor *δ*_*i*_ for each city *i*, defined as

$$\begin{array}{c} \hat{N_{i}}=N_{i}e^{\delta_{i}}, \end{array}$$ (3)

where $\hat{N_{i}}$ is the true resident population, and *N*_*i*_ is an estimated number, such as those provided by Chinese National Statistics. Substituting $\hat{N_{i}}$ into Eq (1) leads to

$$\begin{array}{c} Y_{i}\left(\hat{N_{i}}\right)=Y_{0}{\hat{N_{i}}}^{\beta }e^{-\beta \delta_{i}+\xi_{i}}=Y_{0}{\hat{N_{i}}}^{\beta }e^{\hat{\xi_{i}}}. \end{array}$$ (4)

If we now assume that the scaling law for the actual population is strictly valid for China, so that $\hat{\xi_{i}}=0$ holds, the rescaling factor *δ*_*i*_ can be written as

$$\begin{array}{c} \delta_{i}=\frac{\xi_{i}}{\beta }. \end{array}$$ (5)

Inserting this expression into Eq (3) gives us the true resident population $\hat{N_{i}}$ in terms of observed population, the exponent *β* and the deviation *ξ*_*i*_.

$$\begin{array}{c} \hat{N_{i}}=N_{i}e^{\xi_{i}/\beta }. \end{array}$$ (6)

Eq (6) can now be used to estimate the actual resident population in each city. Cities with positive *ξ*_*i*_ will have new estimated populations larger than those given, and vice-versa.

**Fig 6:**
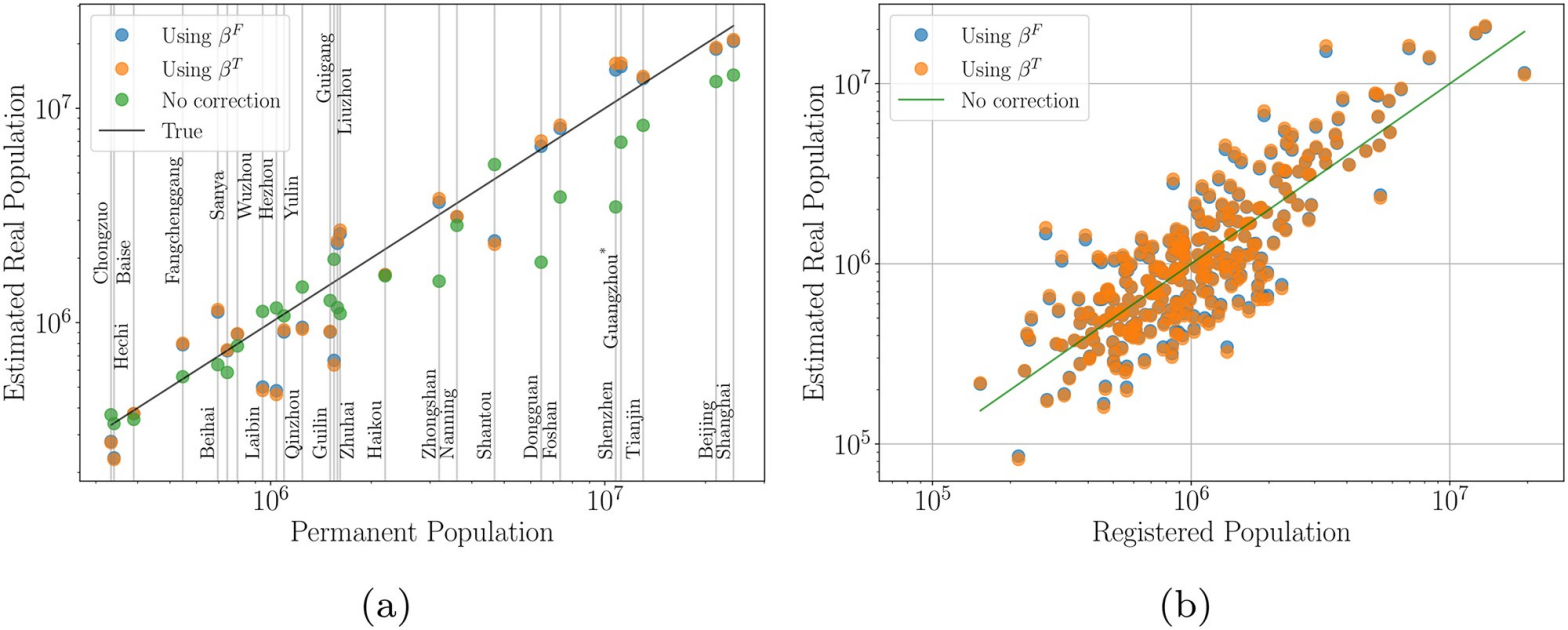
Correction of population city sizes relative to given registered population numbers using the prefecture-level Gross Domestic Product of each city as estimator. *β*^*F*^ is the scaling factor derived by the data and *β*^*T*^ from theory. Comparison of the method with real data for prefecture-level cities that provide data about their true population (a). Corrected city sizes for all prefecture-level cities (b). (*) Actual population sizes for Guangzhou is not available from the China City Statistical Yearbooks, but provided by Guangzhou International [25].

Our exploration of the dataset provided by the China City Statistical Yearbooks shows that aggregated quantities, such as the GDP provide the best estimates, especially for larger cities, as depicted in Fig 6a. The actual population in Beijing is about 21.6 million in 2014 with a registered population (with local Hukou status) of only 13.3 million! Our method corrects this value to 18.8 million when using the *β*^*F*^, the exponent derived from the best fit, and to 19.2 million with *β*^*T*^ from theory (this is the simplest expected value for the exponent from theory, *β*^*T*^ = 7/6). Similarly other good estimates are derived for the largest cities for which data about the permanent population is available. These results are shown in Table 1a for Shanghai, Beijing, Tianjin, Shenzhen, Guangzhou, Foshan, and Dongguan.

**Table 1:** Estimated population sizes for prefecture-level cities in millions for which data about the resident population is available for 2014 and Guangzhou, the largest city in the Pearl River Delta. The Gross Domestic Product is used as the estimator, see text. The corresponding values are plotted in Fig 6a. It is clear that the true population size of large coastal cities is not well described by the Hukou system count, but better captured by the estimates resulting from the residuals of the scaling relations for GDP.

City	Resident Population	Hukou Population	Estimate *β*^*F*^	Estimate *β*^*T*^
Shanghai	24.26	14.29	20.54	20.93
Beijing	21.56	13.33	18.88	19.24
Tianjin	13.02	8.33	13.76	14.09
Guangzhou	11.17*	6.95	15.64	16.24
Shenzhen	10.78	3.47	15.10	16.20
Foshan	7.35	3.86	8.06	8.35
Dongguan	6.45	1.91	6.65	7.05
Shantou	4.68	5.47	2.41	2.32
Nanning	3.61	2.84	3.12	3.13
Zhongshan	3.19	1.56	3.65	3.79
Haikou	2.20	1.65	1.67	1.68
Zhuhai	1.61	1.10	2.60	2.70
Liuzhou	1.58	1.18	2.34	2.42
Guigang	1.55	1.97	0.67	0.63
Guilin	1.51	1.27	0.90	0.91
Qinzhou	1.24	1.47	0.95	0.93
Yulin	1.10	1.08	0.90	0.93
Hezhou	1.04	1.17	0.48	0.46
Laibin	0.95	1.13	0.50	0.48
Wuzhou	0.80	0.78	0.88	0.89
Sanya	0.74	0.59	0.74	0.75
Beihai	0.69	0.64	1.12	1.15
Fangchenggang	0.55	0.57	0.79	0.80
Baise	0.39	0.35	0.38	0.38
Hechi	0.34	0.34	0.23	0.23
Chongzuo	0.33	0.37	0.28	0.28

*Source: Guangzhou International.

For smaller cities, i. e. those with resident population smaller than about 5 million, the same method nudges the population in the right direction, but tends to create corrections that are probably too large.

Smaller cities whose population are underestimated by our proposed method are mostly places located away from the coast. For example, Guilin that has an actual population of 1.5 millions and a registered population of 1.3 millions, is estimated to have a population of 0.9 millions according to its GDP. Guilin’s economy mostly relies on the primary and tertiary sectors, namely local produce and tourism, which is different from the general economic characteristic of the China’s urban system. Similar patterns are shared by all of the cities that receive estimates that are lower than the actual population. Thus, improved estimates could in principle be generated by taking into consideration economic sectoral composition in each city, together with knowledge of the relationship between employment and value.

The biggest surprise from comparing the estimation method to full population counts is the city of Shantou in Guangdong. It is located on the coast in a rich part of China and it has a big harbor. The local economy is dominated by manufacturing as is true for other cities in the same region. At the same time, Shantou is known for being one of the centers for processing electronic waste and for a recent history of administrative disfunction. Nevertheless, the relatively low GDP of Shantou (170 Billion Yuan) would estimate the total number of permanent residents to be much smaller than the actual population of 4.68 million, specifically, at 2.3 million for *β*^*T*^ and 2.4 million for *β*^*F*^. A property that might be explained by the location of Shantou, as it is not located in the vicinity of the Pearl River Delta with its strong economy, but part of the province of Guangdong. Thus, it faces challenges from the siphon effect of the Pearl River Delta, which has an influence on all cities in the province (see Fig 3c).

When the method is applied to all cities in the dataset, including those for which no actual population number are given, a distinct pattern emerges, as expected from the economic development in the last decades. There are three main clusters of cities, which have a significantly higher population compared to the population given by the Hukou system. They are all along the coastline, located around Beijing and Tanjing, Shanghai, and the Pearl River delta.

In inland China, some cities with a small number of people as counted by local Hukou are estimated to have a much larger population according to their GDP. These are mostly cities that are rich in natural resources, and are among the cities with the highest GDP per capita. In such cases, again, taking into account sectoral composition and the translation between productivity and employment may be critical, especially for mining and oil and gas industries.

The nominally largest city in China, Chongqing, is the only large city for which the estimator predicts a smaller population size than the one given by the Hukou counts. As mentioned earlier, the current definition of Chongqing as a direct-controlled municipality leads to an enormous urban area, which is likely a poor approximation for the size and extend of its actual functional city.

In summary, we proposed method to estimate the true resident population of Chinese cities based on deviations from expected scaling (agglomeration) effects within the urban system. The method works reasonably well for larger cities and helps identify if the population of any city is under- or overestimated by the Hukou registration counts. The strength of our method compared to other estimators, such as morphological, big data, remote sensing approaches, or combinations of them [26–30], is that it only needs a few data points to estimate true populations with only a minor loss of precision (see S2 File). Economic sectoral compositions and corresponding relationship between employment and value, if they are known, may generate even better estimates. The method developed here also provides a systematic way to flag cities with economic make ups that deviate from what is expected for China not only in terms of an averages across the system, but by taking city size (and thus agglomeration) into account.

## Discussion

In recent decades, China has undergone one of the most extraordinary historical transformations from a predominantly rural and poor country to one of the world’s biggest economies, the largest exporter and a huge dynamic nation creating rising living standards for its vast population within the context of rapid urbanization and the advent of many large and glitzy cities.

As we write, this transformation remains incomplete and the relation of city sizes to different quantities in the aggregate understudied. Both extant data quality issues and the lack of functional urban area definitions make the present study of Chinese cities necessarily a very approximate exercise, as we discussed throughout this paper. Nevertheless, we have shown here, using available official data for sets of urban districts in Chinese prefecture-level cities, that much of the urbanization of China has close parallels in other nations both past and present. This is remarkable given the level of ambition and impact of government policies in China over the last seven decades and speaks clearly to the extraordinary importance of taking a fundamental scientific perspective regarding the essential properties of cities and of processes of development.

Secondly, a fundamental set of characteristics connected to both the creation and growth of cities and their economies are scaling (agglomeration) effects. These effects express non-linear increases in socioeconomic productivity, as well as savings in infrastructure, with city population size. We have shown that scaling relations for aggregate GDP and land area for the urban portions of China’s prefecture level cities show exponents (elasticities to population size) in line with other nations and with urban scaling theory; indicating quantitatively consistent superlinear effects in economic productivity and sublinear economies of scale in land uses (densification). This is remarkable because the explanation for these effects in terms of urban scaling theory [20] (and analogous approaches in urban economics [31] derives these exponent values from modeling cities as self-organizing socioeconomic networks embedded in built up spaces. In contrast, one could argue that the urban economies of China are much more planned and much more dominated by large scale manufacturing and exports than cities elsewhere, such that general expectations for scaling and agglomeration effects may not apply. Such *Chinese exceptionalism* does not hold empirically, as we showed here. The only potential signal for some of these peculiar characteristics of Chinese urbanization may be found in larger levels of variation away from scaling across places. Such variations may express stronger local contexts and interventions in distinct cities and regions (e.g. Shenzhen), but are certainly also partially the result of statistical uncertainties in data collection and organization.

A couple of features of these results stand out, starting from the fact that the exponent for GDP tends to be slightly larger than predicted by theory (*β*_*GDP*_ ≃ 1.22 ± 0.05 > 1.17) and has greater uncertainty than in other nations. This expresses the fact that Chinese data are much noisier than, say, for the US urban system and, in addition, that a number of large Chinese cities are still under-predicted by the scaling relation. In plain language, these results mean that, when comparing two cities in China one with the double the population of the other, the former will have on average a GDP per capita that is 22% larger, but that this average expectation is less predictive than in other nations. Moreover it says that this number underestimates the actual GDP of some of China’s most prominent cities, such as Shanghai, Beijing, Shenzhen or Tianjin.

We explored the systematic pattern in these deviations from scaling, in the last section, to show how to predict the actual resident population of these cities, instead of the more commonly reported number based on China’s household registration system (Hukou). We showed that, where estimates are available, scaling makes good predictions for the actual total numbers counted. Thus, it is at least plausible that some of the present uncertainty in urban indicators in Chinese cities is related to various inadequate and inconsistent ways to estimate their working populations as well as their economic productivity and costs. It is important that such measurements become more consistent, better aligned with standard indicators in other cities and urban systems, and more spatially disaggregated (down to neighborhood scales, such as census tracts) so that issues of distribution and statistics can be better assessed and understood.

China and its cities face a number of challenges going forward that would strongly recommend a much more systematic scientific analysis of their structural characteristics and patterns of change. As a consequence of China’s historical demographic policies and more recent migration restrictions, many cities in China will likely very soon experience labor shortages and population contractions. These trends will tend to intensify over time as China’s population ages quickly [1]. Given their dependence on exports, and on a logic of strongly planned growth based on infrastructure expansion, these fast systemic trends will likely be met by some structural rigidity, which may result in challenging transitions for the nation and for each city in turn.

Moreover, a transformation to more sustainable cities, economically and environmentally, will need to be quickly achieved—within a couple of decades—based on rising quality of life for urban dwellers, and on improved and more valuable services and innovation, relying on the expanded capabilities of an older, more skilled population. Success at this critical juncture requires not only better and more adaptive policies, but also a more fundamental, comprehensive and evidence-based understanding of China’s rapid urbanization, which will continue to be critical for our global sustainability transition affecting every person in China as well as the planet at large.

## Supporting information

S1 File Patterns of national economic development. (PDF)

S2 File Comparison of population estimates to a morphological definition of Chinese urban areas (ChinaCities). (PDF)
