# Supplementary material for: Growth and development in prefecture-level cities in China
Source: PLoS One. 2019 Sep 3;14(9):e0221017. doi: 10.1371/journal.pone.0221017 (PMC6719855; doi:10.1371/journal.pone.0221017)
Supplement: S1 File — (PDF) [file pone.0221017.s001.pdf]

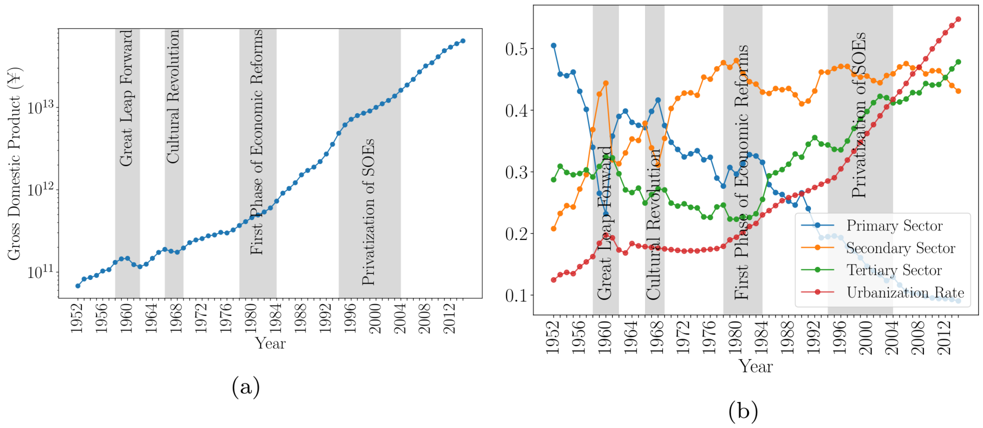

(a)

(b)

S1 Fig. Temporal evolution of China's nominal Gross Domestic Product (a) and the three economic sectors as fraction of GDP (b), together with the nation's urbanization rate. Periods of major economic and political reforms are shown as grey bars. The impact of these reforms on the whole economy and its structure are clearly visible, as well as the long-term shift from an agriculturally dominated economy to an industrialized country, with large secondary and tertiary sectors. Note that only in recent years has the tertiary sector - typically dominant in high income nations - surpassed the secondary in total value.

S1 Patterns of National Economic Development

The unprecedented economic success of China over the two decades discussed in this manuscript has been preceded by a longer history of top-down economic reforms motivated principally by strong ideological motives. These interventions have certainly left a mark in China's history of urbanization and development. Nevertheless, most general characteristics of cities and national patterns of economic development observed in other nations also apply to China over this period.

Motivated by consistent data availability from Chinese national statistics we start our analysis around 1950, shortly after the advent of the People's Republic of China (PRC). Before the first big economic reform in the late 1950s, the Chinese economy was strongly dominated by the primary sector, which accounted for more than half of the nations Gross Domestic Product in value (S1b Fig). Not surprisingly, the country was mostly rural, characterized by a stunningly low urbanization rate of about 12%. The country was very poor compared to industrialized nations, with a GDP per capita thirty times smaller than in United States [1].

Enormous top-down changes of the Chinese economy and society came about with the first comprehensive economic reform in the history of the PRC, at the end of the 1950s, the *Great Leap Forward*. The aim of the reform was to quickly transform the national economy from its focus on the primary sector to an industrialized system.

The immediate change in the nation's economic structure is clearly visible in S1 Fig. China's GDP visibly rises initially, there is also a plummeting drop in the primary sector (from 36% to about 23%) and a corresponding rise of the secondary sector (from 30% to almost 45%), signaling much higher industrialization levels within only a few years (S1b Fig). These transformations were also initially accompanied with a small uptick in China's urbanization rate, to about 20% by 1960.

However, as is well known, these initial transformations could not be sustained and effects were reversed within only a few years. One of the main problems was that, due to rapid change and loss of workforce, the primary sector experienced a big drop in

productivity. This led to a severe under-production of basic goods and to dire shortages, including widespread malnourishment of the population. After several years of a steady decreases in the population's mortality rate prior to the reform, the Great Leap Forward led to a significant increase [2], and eventually to a general economic regression. These catastrophic unintended consequences lead to a complete reversal of statistical trends by 1964, with sector compositions, GDP levels and urbanization rates all reverting to their levels circa 1958.

After the reforms of the Great Leap Forward were abolished, the Chinese economy started to recover slowly, showing some increase in GDP and growth of the secondary sector. The next comprehensive policy, the *Cultural Revolution* started in 1966, and was officially declared as ended by 1969, however, carried on until the mid 1970s. Though it was less directly targeted at the economy, it had several major related effects in suppressing human capital and many aspects of urban life and culture and, reversing a trend of the *Great Leap Forward*, actually increasing labor participation in the primary sector. Correspondingly, the onset of the reform led to a drop in national GDP, and urbanization rate and a decrease in the value of the secondary and tertiary sectors of the economy. The trajectories in S1b Fig show that the secondary sector was impacted most adversely by the reform, which is mainly responsible for the drop in GDP during this time.

The foundations for the economic success of the last two decades were laid shortly after the Cultural Revolution and, in many ways, as a reaction to it. In a first phase of economic reforms starting around 1979, the national government opened up the economy to some foreign investments and allowed its citizens to start their own businesses. Surprisingly, this led at first to a restructuring of the economy that might not be have been expected. The contribution of the secondary sector to GDP decreased initially, while the primary sector gained momentum. This goes back to the experience of the Chinese government from the Great Leap Forward, when labor was diverted from agriculture to the secondary sector and the production level of the primary industry was too low to nourish the country. Thus, government reform policies, at first, placed a focus on changes in the primary sector, which increased its output significantly and accounts for the main fraction of the growth in the early 1980s.

When the primary sector reached a level of productivity that was high enough to sustain the country, reforms were gradually extended to other sectors. As the temporal trajectories show in S1b Fig, the tertiary sector actually benefited first. The possibility for people to start their own businesses increased economic entrepreneurship and is strongly connected to the economic growth and increase of the urbanization rate seen by the late 1980s. It is interesting to see that the secondary sector did not react quickly to these economic reforms and even experienced a slight decrease of its relative contribution to GDP. Later, the fraction of the secondary sector increased again, when it shifted its focus from raw materials to more complex products, such as garments and electronics [3], mainly for export.

This shift of the secondary sector to the production of more complex tradable goods coincides with the last big change of China's economic structure. Starting in the early 1990s, the government started to privatize most of the state-owned enterprises (SOEs) [4]. At first, the reform decelerated the growth of the economy and led to a massive layoff of workers [5], which resulted in turn in an increase of the tertiary sector in terms of contribution to the GDP, and a decrease of the secondary sector overall. However, the higher efficiency eventually gained by reforming former SOEs ultimately strengthened the secondary sector, making it possible to shift their focus to more complex products, and, together with the accession of China to the World Trade Organization, arguably laid the foundation for the large growth rates of the Chinese economy after the year 2000 [6, 7].

The specificity of Chinese policies notwithstanding, this sector rotation is typical of patterns of national and regional growth and urbanization in many nations. The biggest difference in the development of the Chinese economy compared to other historical examples is the speed of change and the extreme nature of government interventions. As a comparison, it took Japan's economy 80 years to develop a similar economic sector make up to its GDP, which China reached within a little more than two decades [8]. In terms of GDP value, the Japanese economy reached the second highest GDP in the world at the end of the 20th century, a ranking it held till 2009. By 2014, China's GDP was already double that of Japan's and is presently quickly catching up to the US's total GDP. In 1980, China's Gross Domestic Product was only about 7% of that of the USA. By 2014, it had increased to be more than 60% [1].

In tandem with these macroeconomic trends, there has also been a substantial change in people's livelihoods. The disposable income of households grew in a similar rate to GDP [9], increasing by a spectacular factor of almost one hundred since the 1970s in nominal terms (about ten times in real, inflation adjusted terms). The additional disposable incomes of households is one of the main reason for the growth of the tertiary sector, as it generally implies an expanding demand for services.

Historically, such changes tend to result in the growth of services and eventually, in a historical context, create a post-industrialized economy. S1b Fig suggests that China is presently in the process of shifting from an industrializing country to a post-industrial, services-based nation at least as far as the value fraction of GDP is concerned. However, the high contribution of the secondary sector to GDP has been surprisingly stable under this assumption: ever since the Cultural Revolution the share of the secondary sector has remained above 40%. Compare for GDP share of the tertiary sector in the United States, which has always been higher than the share of the secondary sector since data are available. For example, already in 1948 the private tertiary sector accounted for 47% of the US's GDP, whereas the private goods-producing sectors contributed 41%. This gap steadily increased since then and presently stands at about 67% and 20%, respectively [10]. A possible explanation for the difference in the sectoral composition might suggest that the trajectory of the Chinese economy is unlike the trajectory of today's advanced societies [11], in which a manufacturing plays a stronger role [12,13].

Another possible explanation, which is more in line with historical examples, for this modest fraction of the tertiary sector in China is its broad definition according to official statistics. Available data shown in S1b Fig lumps together highly specialized services requiring high levels of human capital and creating high value-added, such as healthcare or finance, with more mundane businesses, such as small restaurants or cleaning services. The high diversity of services in the tertiary sector and the trajectories of those sectors in S1b Fig suggest that most of the labor force in the tertiary sector was previously working in the primary sector. For a variety of reasons, which include obstacles to access to education and training for populations with a rural Hukou, such workers will initially possess lower average human capital and thus be able to only generate lower value-added services, requiring less specialization.

Because of all these factors, diverse cities in China show different patterns of growth and development and different sector compositions, from common domination of the secondary sector to some cases where primary activities are still important, and to more cosmopolitan megacities, where a transition to a service based economy is also already well under way. S2 Fig depicts the top 275 (prefecture-level) cities in terms of their population size in China.

References

1. The World Bank; 2018. Available from: `https://data.worldbank.org`.

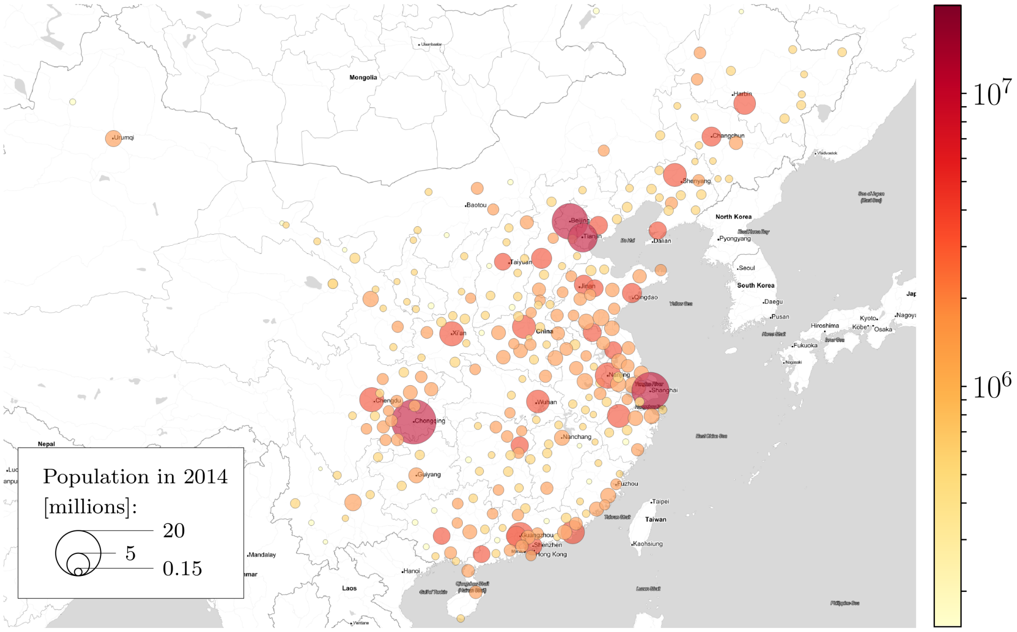

S2 Fig. Spatial distribution of the 275 largest Chinese prefecture-level cities [14]. The area of the symbols and the color coding is scaled in terms of registered population. Most of the largest cities in China are located close to the coast, benefiting from access to international transportation routes.

2. Peng X. Demographic consequences of the Great Leap Forward in China's provinces. Population and development review. 1987; p. 639–670.

3. The Observatory of Economic Complexity; 2018. Available from: `https://atlas.media.mit.edu/`.

4. Lu M, Fan J, Liu S, Yan Y. Employment Restructing during China's Economic Transition. Monthly Lab Rev. 2002;125:25.

5. Liu H, Zhao Z. Parental job loss and children's health: Ten years after the massive layoff of the SOEs' workers in China. China Economic Review. 2014;31:303–319.

6. He C, Wang J. Regional and sectoral differences in the spatial restructuring of Chinese manufacturing industries during the post-WTO period. GeoJournal. 2012;77(3):361–381.

7. He C, Wang J. Does ownership matter for industrial agglomeration in China? Asian Geographer. 2012;29(1):1–19.

8. Economic History Association; 2017. Available from: `https://eh.net`.

9. Stratford K, Cowling A. Chinese Household Income, Consumption and Savings. RBA Bulletin,(September). 2016; p. 31–40.

10. Bureau of Economic Analysis; 2018. Available from: `https://www.bea.gov`.

11. Rodrik D. Premature deindustrialization. Journal of Economic Growth. 2016;21(1):1–33.

12. Solo RA, Zysman J. Stephen S. Cohen and John Zysman: Manufacturing Matters; The Myth of the Post-Industrial Economy (Book Review). Journal of Economic Issues. 1988;22(3):880.

13. Walker RA. Is there a service economy? The changing capitalist division of labor. Science & Society. 1985; p. 42–83.

14. Map tiles by Stamen Design, under CC BY 3 0 Data by OpenStreetMap, under ODbL; 2018.
