# Supplementary material for: Growth and development in prefecture-level cities in China
Source: PLoS One. 2019 Sep 3;14(9):e0221017. doi: 10.1371/journal.pone.0221017 (PMC6719855; doi:10.1371/journal.pone.0221017)
Supplement: S2 File — (PDF) [file pone.0221017.s002.pdf]

S2 Comparison of Population Estimates to a Morphological Definition of Chinese Urban Areas (ChinaCities)

In addition to the official prefectural-level definitions of urban areas used in the main text and several other studies [1–4], there have been recent efforts to construct delineations of urban areas in China based on morphology [5–9]. Here, we compare population estimates following from the two different methods by comparing it with the estimates from ChinaCities [7].

S3 Fig and S1 Table show the population estimates according to our estimated population and the morphological definitions in ChinaCities. The diagonal line represents perfect agreement. We see that most large cities have similar population estimates in both data sets. For smaller cities, some of the estimates disagree, with ChinaCities predicting typically smaller populations. This might arise from the methodology used in ChinaCities, which separates different cities and towns within the same prefecture-level city, if there is no continuous built fabric between them. The threshold to delineate two cities as separate is a 200 meters gap in built fabric, judged on the basis of satellite imagery from the year 2000 with a resolution of 2 meters. Thus, when two agglomerations are further apart from each other than 200 hundred meters, they are counted as two different cities [7]. This means that where we regard the whole administrative unit (urban districts) as one city, ChinaCities' methodology might split it up or extend it based on the shape (morphology) of built areas. A good example is Chongqing's prefecture-level city, whose size is similar to Austria's and is nominally the largest city in China according to official statistics. This is not a good definition of the functional city, which is likely significantly smaller [10]. Both, the morphological and our population estimate using GDP, predict similar true population for Chongqing. Future research should focus on improving functional definitions in China with special care on the integration of their economies and labor markets, beyond morphological and administrative units. As the comparison of this section makes clear, the main challenge deals with adopting functional urban definitions that apply also to smaller cities where built environment and population densities may be lower, but where commuting ties may (or may not) keep extended areas integrated.

References

1. Rohde RA, Muller RA. Air pollution in China: mapping of concentrations and sources. PloS one. 2015;10(8):e0135749.

2. Fang LQ, de Vlas SJ, Liang S, Looman CW, Gong P, Xu B, et al. Environmental factors contributing to the spread of H5N1 avian influenza in mainland China. PloS one. 2008;3(5):e2268.

3. Zhao J, Chen S, Jiang B, Ren Y, Wang H, Vause J, et al. Temporal trend of green space coverage in China and its relationship with urbanization over the last two decades. Science of the Total Environment. 2013;442:455–465.

4. Tao R, Su F, Liu M, Cao G. Land leasing and local public finance in China's regional development: Evidence from prefecture-level cities. Urban Studies. 2010;47(10):2217–2236.

5. Long Y. Redefining Chinese city system with emerging new data. Applied geography. 2016;75:36–48.

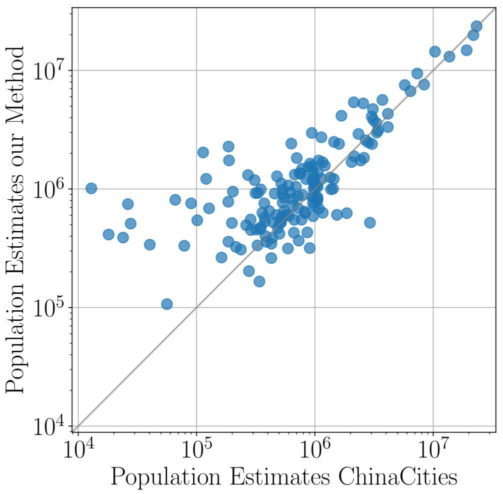

S3 Fig. Comparison of estimated populations by the method introduced in the manuscript to a morphological definition of Chinese cities (ChinaCities) [7]. Cities are shown as blue circles; 154 urban areas were matched based on names and geographic location between the two data sets. The diagonal solid line shows one-to-one agreement, for reference. We find good agreement between the two approaches for large cities. For smaller cities, some population estimates disagree, presumably because urban areas were decomposed into separate cities according to their morphological definition. Future studies seeking to construct improved functional urban area definitions for China (integrated labor markets) should seek to resolve these discrepancies.

S1 Table. Population sizes of 20 cities that could be reasonably well matched between prefecture-level data and ChinaCities for 2010 [7]. The main differences between the estimates comes from the different methodologies used in the methods. We used the matched data set and GDP to derive $\beta^F = 1.16$, which is smaller than $\beta^F = 1.19$ for the full data set for prefecture-level cities.

City	Hukou Population	ChinaCities	Estimate β^F	Estimate β^T
Shanghai	13.43	23.26	23.57	21.23
Beijing	11.90	21.86	19.85	17.89
Guangzhou	6.64	19.16	14.78	13.35
Tianjin	8.11	13.71	13.06	11.81
Shenzhen	2.59	10.35	14.40	13.00
Wuhan	8.36	8.37	7.58	6.88
Chongqing	15.60	7.33	9.41	8.52
Chengdu	5.35	6.43	6.68	6.06
Nanjing	5.48	5.74	7.52	6.82
Zhengzhou	2.93	4.14	3.32	3.03
Changchun	3.62	4.13	4.30	3.91
Qingdao	2.75	3.71	5.63	5.12
Taiyuan	2.85	3.42	3.11	2.83
Kunming	2.50	3.33	2.99	2.72
Hefei	2.15	3.31	3.60	3.27
Xiamen	1.80	3.11	3.82	3.48
Changsha	2.42	3.09	4.71	4.29
Wenzhou	1.45	3.03	2.39	2.18
Zibo	2.79	3.02	4.07	3.71
Fuzhou	1.12	2.92	0.51	0.47

6. Cheng L, Zhou Y, Wang L, Wang S, Du C. An estimate of the city population in china using dmsp night-time satellite imagery. In: Geoscience and Remote Sensing Symposium, 2007. IGARSS 2007. IEEE International. Ieee; 2007. p. 691–694.

7. Swerts E. A data base on Chinese urbanization: ChinaCities. Cybergeo: European Journal of Geography. 2017;.

8. Song Y, Long Y, Wu P, Wang X. Are all cities with similar urban form or not? Redefining cities with ubiquitous points of interest and evaluating them with indicators at city and block levels in China. International Journal of Geographical Information Science. 2018;32(12):2447–2476.

9. Chen Y, Wang J, Long Y, Zhang X, Liu X, Li X. Defining Urban Boundaries by Characteristic Scales. arXiv preprint arXiv:171001869. 2017;.

10. Jia G, Xu R, Hu Y, He Y. Multi-scale remote sensing estimates of urban fractions and road widths for regional models. Climatic Change. 2015;129:543–554. doi:10.1007/s10584-014-1114-3.
